# *Salmonella* Enteritidis Infections Associated with Foods Purchased from Mobile Lunch Trucks — Alberta, Canada, October 2010–February 2011

**Published:** 2013-07-19

**Authors:** Lance Honish, Dawn Greenwald, Kristin McIntyre, Wendy Lau, Sarah Nunn, Dale Nelson, Judy MacDonald, Victoria Keegan, Krista Wilkinson

**Affiliations:** Alberta Health Services; Canadian Field Epidemiology Program, Public Health Agency of Canada

During October 2010–February 2011, an outbreak of 91 *Salmonella* Enteritidis (SE) infections in Alberta, Canada, was investigated by a local public health department (Alberta Health Services, Calgary Zone). Index cases initially were linked through a common history of consumption of food purchased from mobile food-vending vehicles (lunch trucks) operating at worksites in Alberta. Further investigation implicated one catering company that supplied items for the lunch trucks and other vendors. In 85 cases, patients reported consumption of food prepared by the catering company in the 7 days before illness. Six patients were employees of the catering company, and two food samples collected from the catering company were positive for SE. Foods likely were contaminated directly or indirectly through the use of illegally sourced, SE-contaminated eggs at the implicated catering facility and by catering employees who were infected with SE. Public health interventions put into place to control the outbreak included screening employees for *Salmonella,* excluding those infected from food-handling duties, and training employees in safe food-handling procedures. No further outbreak cases were identified after full implementation of the interventions. This investigation highlights the potential for lunch trucks to be a source of foodborne illness and the need for robust regulatory compliance monitoring of lunch trucks and their food suppliers.

## Epidemiologic Investigation

The case definition for this outbreak was a laboratory culture-confirmed SE infection identified in Alberta during October 2010–February 2011 that was epidemiologically linked to the implicated catering firm. The 91 outbreak cases were in customers of lunch trucks (78), gas stations (three), or vending machines (two) that received product from the implicated caterer. Six were employees of the implicated caterer, and two were lunch truck drivers. Patients resided in Calgary (86), metro Edmonton, (three), and southern Alberta (two); all resided in different households. Lunch truck customer cases were among employees of several different workplaces. Median age at onset of symptoms was 32 years, with a range of 19 to 68 years; 76% of cases were among males. Reported signs and symptoms included diarrhea (96%), abdominal cramps (85%), fever (52%), bloody diarrhea (33%), and vomiting (25%); two patients, both employees of the implicated catering company, were reportedly asymptomatic and detected by screening. Six patients (7%) were hospitalized. Illness onset dates ranged from October 1, 2010, to February 14, 2011 (Figure). Human SE outbreak isolates were phage type 8 (49 [54%]), 13 (32 [35%]), or atypical (10 [11%]). Of the 1,311 human SE isolates reported in Alberta during 2006–2010, the proportion that were phage type 8, 13, and atypical was 30%, 11%, and 3%, respectively (Alberta Health Services, unpublished data, 2011).

Most lunch truck customer patients (57%) could not recall the specific vehicle from which they purchased food, resulting in positive identification of only 14 lunch trucks. Patient food histories included more than 40 different lunch truck food items; those most frequently reported were breakfast egg sandwiches (38%) and pork dumplings (24%). Patients often reported consuming multiple meals from lunch trucks during the incubation period.

## Environmental Investigation

Local public health department environmental health officers (EHOs) located 54 lunch trucks in operation for targeted inspections. All food received by trucks was prepackaged; perishable food was refrigerated and/or reheated in the vehicle. Several violations were observed, including selling foods from unlicensed food facilities and inadequate reheating of previously cooked food.

Seven Calgary-based caterers were identified as lunch truck suppliers; all had been routinely inspected by the public health department. Eggs at several catering facilities were found to be from unapproved sources and were ungraded, cracked, visibly dirty, and/or improperly packaged, resulting in a parallel investigation of the egg suppliers. Ongoing traceback of food items consumed by persons infected in the outbreak implicated one caterer as the source of illnesses. A bucket used for mixing pooled eggs by the implicated caterer during preparation of breakfast sandwiches had not been cleaned for several weeks and was stored in a cooler between uses. Pork dumplings sold by lunch trucks were produced by a Calgary-based manufacturer, and approximately 1,000 were distributed uncooked and frozen to several caterers each day. Caterers cooked, packaged, and refrigerated the dumplings before distribution to lunch trucks. Eggs were reportedly not an ingredient of the dumplings. The pork dumpling cooking procedure used by the implicated caterer was deemed adequate by EHOs.

A total of 32 food samples were collected from lunch truck suppliers. Two food samples collected at the implicated catering facility were culture positive for SE: 1) pork dumplings received raw and frozen and subsequently cooked and packaged by the implicated caterer (SE phage type 13) and 2) a sample of raw egg mixture from the egg bucket described previously (SE phage type 8). All other samples collected during the investigation were *Salmonella* culture negative, including raw and cooked pork dumplings collected at the place of manufacture. Numerous sanitation, food-handling, and employee hygiene violations were identified.

## Actions Taken

Employees of the implicated caterer were screened for *Salmonella* in stool (initially and in three successive rounds later in the outbreak) as required by the local Medical Officer of Health; stool specimens from six of 14 employees were positive for SE. The symptom onset dates of the four symptomatic infected employees ranged from November 1, 2010, to January 15, 2011. The infected employees prepared food as part of their duties and were excluded from food-handling duties by order of the Medical Officer of Health until deemed noninfectious. Employees also were screened at the pork dumpling manufacturing facility and selected catering facilities known to have supplied lunch trucks during the outbreak; all were negative for SE.

The pork dumpling manufacturer was required to label the product with cooking instructions (i.e., cook to an internal temperature of 71°C [160°F]), and EHOs ensured that these instructions were followed by recipient caterers before product distribution. Additional interventions at the implicated catering facility included provision of on-site, safe food-handling employee training by the local health department (in the first language of employees), a thorough cleaning and disinfection of the catering facility and equipment by a contracted company, and implementation of an EHO-approved food safety plan developed by a third-party consultant. Voluntary, safe food-handling training courses were provided to lunch truck operators by EHOs. No further outbreak cases were identified after full implementation of the interventions.

Despite intensive monitoring of the implicated caterer and apparent confirmation that all eggs used were from legal sources, illegally sourced eggs were discovered at the facility after the outbreak, resulting in prosecution of the caterer for breaching Alberta public health legislation. An illegal egg supplier identified through this investigation also was charged. Several thousand eggs were seized from the supplier, and subsequent enforcement actions resulted in seizure of an egg delivery vehicle, issuance of a $2,500 fine, and incarceration of the supplier for 14 days.

What is already known on this topic?

Eggs are an important vehicle for the transmission of *Salmonella* Enteritidis to humans.

What is added by this report?

An outbreak of 91 *Salmonella* Enteritidis infections was linked to a catering company that supplied food items for lunch trucks and other vendors. Foods likely were contaminated directly or indirectly through the use of illegally sourced eggs or by infected catering employees. No further outbreak cases were identified after full implementation of public health interventions, which included training in and enforcement of approved food-handling procedures.

What are the implications for public health practice?

This investigation highlights the potential for lunch trucks to be a source of foodborne illness and the need for robust health department inspections of lunch trucks and their suppliers.

### Editorial Note

In Canada, approximately 6,000 laboratory-confirmed cases of *Salmonella* are reported annually, with Enteritidis the most frequently reported serotype (32.1% of isolates in 2009) (1). Incidence rates in the Calgary area typically are comparable to those observed nationally (Alberta Health Services, unpublished data, 2011). The source of this outbreak likely was a catering firm that supplied items for lunch trucks throughout Calgary. Foods likely were contaminated through the use of SE-contaminated eggs obtained from unapproved sources, subsequent cross-contamination through improper food-handling practices, and handling of food by SE-infected employees.

Eggs have been established as an important vehicle for human SE infections. The use of ungraded, illegally distributed eggs was a possible factor in a recent large SE cluster in British Columbia (2), and pooling of eggs and cross-contamination of food contact surfaces was a factor in other SE outbreaks (3). Consistent with other outbreaks associated with foodborne *Salmonella* (4), *Salmonella* probably entered the facility via contaminated eggs, with infected food handlers and environmental contamination resulting in transmission to customers. Outbreak SE isolate phage typing data provided an epidemiologic link between food contaminated by the implicated caterer and outbreak cases but do not help to confirm patterns of contamination and transmission. The implicated catering facility was routinely inspected before the outbreak, but new cases ceased only after more intensive review and improvement of food handling practices at the facility, as well as screening of facility employees and exclusion of SE-infected workers.

Prolonged shedding of SE by symptomatic and asymptomatic food workers might have contributed to the duration of the outbreak; median duration of shedding in excess of 30 days has been observed previously in SE-infected food workers (5). The probable ongoing use of illegally sourced eggs and improper handling of eggs by the implicated caterer also might have been a factor in outbreak duration. Proper reheating of pork dumplings by lunch truck operators likely would have prevented some of the outbreak cases. This investigation highlights the need for robust health department inspections of these food facilities and their suppliers.

## Figures and Tables

**FIGURE:**
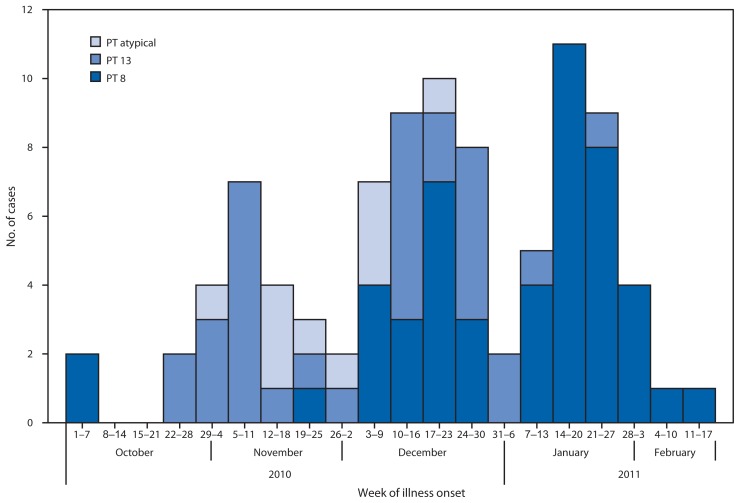
Number of laboratory-confirmed *Salmonella* Enteritidis infection cases (N = 91^*^) epidemiologically linked to the implicated catering firm, by phage type (PT) and week of illness onset — Alberta, Canada, October 2010–February 2011 ^*^ Specimen collection date was used as proxy onset date for two asymptomatic cases.
